# Expression pattern of matrix metalloproteinases in human gynecological cancer cell lines

**DOI:** 10.1186/1471-2407-10-553

**Published:** 2010-10-13

**Authors:** Andrea Schröpfer, Ulrike Kammerer, Michaela Kapp, Johannes Dietl, Sonja Feix, Jelena Anacker

**Affiliations:** 1 Department of Obstetrics and Gynecology, University of Wuerzburg, Josef-Schneider Str. 4, 97080 Wuerzburg, Germany

## Abstract

**Background:**

Matrix metalloproteinases (MMPs) are involved in the degradation of protein components of the extracellular matrix and thus play an important role in tumor invasion and metastasis. Their expression is related to the progression of gynecological cancers (e.g. endometrial, cervical or ovarian carcinoma). In this study we investigated the expression pattern of the 23 MMPs, currently known in humans, in different gynecological cancer cell lines.

**Methods:**

In total, cell lines from three endometrium carcinomas (Ishikawa, HEC-1-A, AN3 CA), three cervical carcinomas (HeLa, Caski, SiHa), three chorioncarcinomas (JEG, JAR, BeWo), two ovarian cancers (BG-1, OAW-42) and one teratocarcinoma (PA-1) were examined. The expression of MMPs was analyzed by RT-PCR, Western blot and gelatin zymography.

**Results:**

We demonstrated that the cell lines examined can constitutively express a wide variety of MMPs on mRNA and protein level. While MMP-2, -11, -14 and -24 were widely expressed, no expression was seen for MMP-12, -16, -20, -25, -26, -27 in any of the cell lines. A broad range of 16 MMPs could be found in the PA1 cells and thus this cell line could be used as a positive control for general MMP experiments. While the three cervical cancer cell lines expressed 10-14 different MMPs, the median expression in endometrial and choriocarcinoma cells was 7 different enzymes. The two investigated ovarian cancer cell lines showed a distinctive difference in the number of expressed MMPs (2 vs. 10).

**Conclusions:**

Ishikawa, Caski, OAW-42 and BeWo cell lines could be the best choice for all future experiments on MMP regulation and their role in endometrial, cervical, ovarian or choriocarcinoma development, whereas the teratocarcinoma cell line PA1 could be used as a positive control for general MMP experiments.

## Background

Tumor invasion and metastasis define malignancy and are the principal causes of cancer associated death. Tumor cells are surrounded by the extracellular matrix (ECM) comprising of proteoglycanes and non-proteoglycanic matrix components (collagen, laminin, fibronectin and elastin). Degradation of the extracellular matrix allows tumor cells to detach from the primary tumor mass, invade local tissue, intravasate, extravasate and build new metastatic formations [1]. Currently, four classes of proteinases are known as being capable of breaking down nearly all components of the extracellular matrix: serine proteinases, aspartatic proteases, cystein proteinases and matrix metalloproteinases (MMPs) [2-4]. Previous studies showed that MMPs facilitate tumor invasion and metastasis in general. Compared to normal tissue, in almost all human cancers the expression and activation of MMPs is increased [5,6]. Also, MMPs play a role in a multiplicity of physiological processes requiring tissue remodeling (e.g. wound-healing, embryogenesis, angiogenesis and ovulation) [2-4]. There is a precise regulation between activation and inhibition of proteolysis and this physiological balance seems to be disrupted in cancer [7].

MMPs are a family of structural and functional related endopeptidases. Currently, 23 members of the MMP family are known in humans [2]. MMPs are zinc dependent proteases which are capable of degrading one or more components of the extracellular matrix. Depending on their substrate specificity, MMPs are divided into six subclasses: collagenases, gelatinases, stromelysins, matrilysins, membrane-type MMPs and others [2]. MMPs are synthesized as inactive zymogens. First they remain inactive by an interaction between the prodomain and the zinc-ion bound to the catalytic site. After removal of the propeptide domain, the active site becomes available to cleave substrates. All MMPs, except MMP-11, are secreted as inactive zymogens and are activated outside the cell by other activated MMPs or serine proteases (e.g trypsin, plasmin, kallikrein) [2-4]. Under physiological conditions, expression of MMPs is tightly regulated on an mRNA level (transcription), e.g. activation of MMPs and inhibition of active MMPs by TIMPs (tissue inhibitors of MMPs).

There is evidence, that the expression of MMPs is related to the progression of gynecological cancers, as is such the case for endometrium cancer [8,9], cervical carcinoma [10-13] and ovarian carcinoma [14-17]. However, only a few MMP-members were investigated in these previous studies. In order to enlarge the knowledge on the role of MMPs plays in these cancer entities, we investigated the expression of all MMPs known in humans so far by measuring mRNA and protein level in twelve gynecological cancer cell lines commonly used in experimental research. We examined cell lines of endometrium carcinoma (Ishikawa, HEC-1-A, AN3 CA), cervix-carcinoma (HeLa, Caski, SiHa), chorioncarcinoma (JEG, JAR, BeWo), ovarian cancer (BG-1, OAW-42) and the teratocarcinoma cell line PA-1.

Until now, only limited data are available on the expression of MMPs in the cell lines investigated herein. Giambernardi and colleagues found the expression of MMP-7, -14, -15, -16 and -17 in HeLa cells on mRNA level as well as an expression of MMP-12 and MMP-14 mRNAs in JEG cell line using RT-PCR [18]. MMP-14 was also detected in the cervix-carcinoma cell lines Caski and SiHa [19]. For an overview, data published so far are summarized in Additional file 1: MMP expression in gynecological cancer cell lines.

## Methods

### Cell culture

All the cell lines used were described in Table 1[20-45] and obtained from Cell Lines Service (Eppelheim, Germany). Briefly, all cells were cultured in a 1:1 mixture of DMEM/Ham's F-12 supplemented with 10% FCS and 10 ng/ml gentamycine (PAA, Coelbe, Germany) at 37°C in the presence of 5% CO_2_. Cells were cultured in 75 ml culture-flasks (Biochrom, Berlin, Germany) as monolayer culture and harvested at 80-90% confluency using a cell-scraper (Biochrom). Cells were resuspended and washed twice in phosphate-buffered saline (PBS). Dry pellets were frozen at -20°C for RNA and protein extraction.

**Table 1:** Human gynecological cell lines

Cell line	Tissue	Cell type	Origin	Special features	Citation
**Ishikawa**	Endometrium	Adenocarcinoma	Primary tumor	ER positive, PR positive	[20-22]

**HEC-1-A**	Endometrium	Adenocarcinoma	Primary tumor	ER positive PR positive	[23-25]

**AN3-CA**	Endometrium	Adenocarcinoma	Metastatic site (lymph node)	ER positive PR positive	[26-28]

**HeLa**	Cervix	Adenocarcinoma	Primary tumor	HPV-18 positive	[29-31]

**Caski**	Cervix	Epidermoid carcinoma	Metastasis (small bowel mesentery)	HPV-16 positive HPV-18 positive	[32,33]

**SiHa**	Cervix	Squamous cell carcinoma	Primary tumor	HPV-16 positive	[34,35]

**JEG**	Placenta	Chorioncarcinoma	Primary tumor	Produce hCG, HCS, progesterone	[36,37]

**JAR**	Placenta	Chorioncarcinoma	Primary tumor	Produce estrogen, progesterone, hCG, HCS	[38]

**BeWo**	Placenta	Chorioncarcinoma	Metastatic site (cerebral metastasis)	Produce estrogen, progesterone, hCG, HCS, estrone, estriol, estradiol, keratin	[38-40]

**BG_1**	Ovary	Adenocarcinoma	Primary tumor	ER positive PR positive	[41,42]

**OAW 42**	Ovary	Cystadenocarcinoma	Metastatic site (ascites)		[43,44]

**PA1**	Ovary	Teratocarcinoma	Metastatic site (ascites)		[45]

### RNA extraction and cDNA synthesis

Total RNA was extracted from 10^6 ^cells using RNeasy mini kit (Qiagen, Hilden, Germany) according to the manufacturer's instruction. Total cellular RNA was eluted in 60 μl RNase free water and stored at -20°C. Total RNA was transcribed at 42°C for 1 h in a 20 μl reaction mixture using the RevertAid H Minus First Strand cDNA synthesis kit (Fermentas, St. Leon-Rot, Germany) and terminated by heating the samples at 70°C for 10 min. Synthesized cDNAs were stored at -20°C for further expression analysis.

### Semiquantitative RT-PCR

Expression analyses of MMPs were performed using gene specific primers and optimized reaction conditions as published previously [46]. Conventional PCR reactions were performed in a volume of 25 μl containing template DNA, 2.5 U Taq polymerase, 10 X reaction buffer with 1.5 mM MgCl_2 _(Eppendorf, Hamburg, Germany), 200 μM dNTPs (Fermentas), 0.4 μM of both forward and reverse primers and formamide at a final concentration of 4%. PCR conditions were optimized for each primer-pair. Amplification reactions were performed using a Px2 thermal cycler (Techne, Staffordshire, U.K.) and consisted of following steps: 94°C for 5 min, 28-32 cycles at 94°C for 30 sec; optimized annealing temperature for 30 sec and 72°C for 10 min (elongation). The amount of cDNA was normalized to the intensity of the PCR products of the ubiquitously expressed gene porphobilinogen deaminase (PBGD). PCR products were separated on a 1% agarose gel and visualized using ethidium-bromide (Roth, Karlsruhe, Germany). All RT-PCRs were performed in independent triplicates.

### Western blotting

For protein extraction, 10^6 ^cells were lysed in precooled Ripa-buffer (Pierce, Rockford, Ilinois) containing phosphatase inhibitors (Phosphatase Inhibitor Cocktails Set II, Calbiochem, Germany), proteinase inhibitors (Complete, Roche, Germany) and 2,5 mM DTT reducing agent (Dithiothreitol, Sigma, Taufkirchen, Germany). The mixture was incubated for 30 min on ice, combined with vortexing every 10 min. Cell lysates were clarified of cell debris by centrifugation at 14.000 × g for 5 min through a QIAshredder spin column assembly (Qiagen) at 4°C. Protein concentration was determined by the Bradford-method [47] using coomassie brilliant blue (Roti-Quant; Roth, Karlsruhe, Germany). Afterwards, the samples were mixed in 5 × loading buffer (Fermentas), denatured at 95°C for 5 min, chilled on ice and stored at -20°C for further analysis. Equal amounts of proteins (20 μg) were loaded on a 10% polyacrilamide gel (SDS-PAGE) and electrophoresed. Proteins were then blotted onto a nitrocellulose membrane (Schleicher & Schuell, Dassel, Germany) for 45 min at 10 V using a semi-dry-transfer unit (PeqLab, Erlangen, Germany). The membrane was stained with ponceau-red (Sigma) to verify that the proteins were blotted. To avoid unspecific binding, the membrane was blocked with 5% nonfat milk in PBS/Tween (0,05%) at RT for 1 hour. Subsequently, the membrane was incubated with the primary antibody at appropriate dilution in 2% nonfat milk and PBS/Tween at 4°C for 18 hours. Primary antibodies used are summarized in Table 2. After washing with PBS, the membrane was incubated with the respective horseradish peroxidase-conjugated secondary antibodies for 60 min at RT. A monoclonal mouse anti-β-actin primary antibody, diluted 1: 10.000, (Abcam, Cambridge, USA) was used as internal control. Immunoblots were visualized by homemade enhanced chemiluminescence (ECL) [48] with subsequent exposure on an X-ray film (Fuji Super RX medical X-ray films; Fuji Photo Film, Duesseldorf, Germany).

**Table 2:** List of antibodies used for Western blot

Gene	Protein forms detected by WB*	Species	Type/clone	Dilution	Company
MMP-1	latent and active	rabbit	polyclonal	1:750	Biozol
MMP-2	latent and active	rabbit	polyclonal	1: 1000	Abcam
MMP-9	latent and active	mouse	9D4.2	1: 500	Chemicon
MMP-11	latent and active	mouse	SL 3.01	1: 500	Abcam
MMP-13	latent and active	mouse	87512	1: 500	R&D
MMP-15	latent and active	rabbit	polyclonal	1: 500	Abcam
MMP-23	latent and active	rabbit	polyclonal	1: 1000	Abcam
MMP-24	latent and active	rabbit	polyclonal	1: 1000	Abcam
MMP-28	not specified	rabbit	polyclonal	1: 1000	Abcam
β-actin		mouse	M/Abcam 8226	1: 10.000	Abcam

*WB: Western blot

### Gelatin zymography

Cell supernatants were collected after 48 hours incubation in serum-free medium. Enzymatic activity of MMP-2 and MMP-9 was measured by gelatinolytic zymography. Conditioned media (20 μl) were incubated with SDS gel sample buffer (Invitrogen, Carlsbad, USA) for 10 minutes at room temperature and electrophoresed on 10% Novex precast zymogram (gelatin) gels (Invitrogen). The gels were run, renatured and developed according to the manufacturer's instructions. Briefly, after electrophoresis, the gels were rinsed twice with Novex Zymogram Renaturing Buffer (30 minutes per wash at room temperature). The gels were then rinsed with fresh Novex Zymogram Developing Buffer and incubated in the same buffer for 18 hours at 37°C. After incubation, the gels were briefly rinsed in distilled water and stained with Coomassie brilliant blue G250 for 2 hours. The digested area appeared clear on a blue background, indicating the expression and activity of gelatinases. The molecular weights of the gelatinases in the samples were determined using recombinant protein molecular weight markers MMP-2 and MMP-9 (R&D Systems, Wiesbaden, Germany).

### Data analysis and statistics

The intensity of ethidium-bromide luminescence and protein expression in Western Blot images was quantified densitometrically using ImageJ image-processing software package (ImageJ: National Institutes of Health, Bethesda, MD, USA), as abovementioned, and normalized in respect to the corresponding fragment concentration of the ubiquitously expressed genes PBGD and β-actin. Four different expression levels were considered in respect of their densitometric value. Value 0 was considered to be no expression. Values between 1 and 19 were considered as very weak ((+)), between 20 and 49 as weak (+), between 50 and 79 as moderate (++) and between 80 and 100 as high (+++) expression.

## Results

### Expression of MMP mRNA in different gynecological cancer cell lines

**Figure 1:**
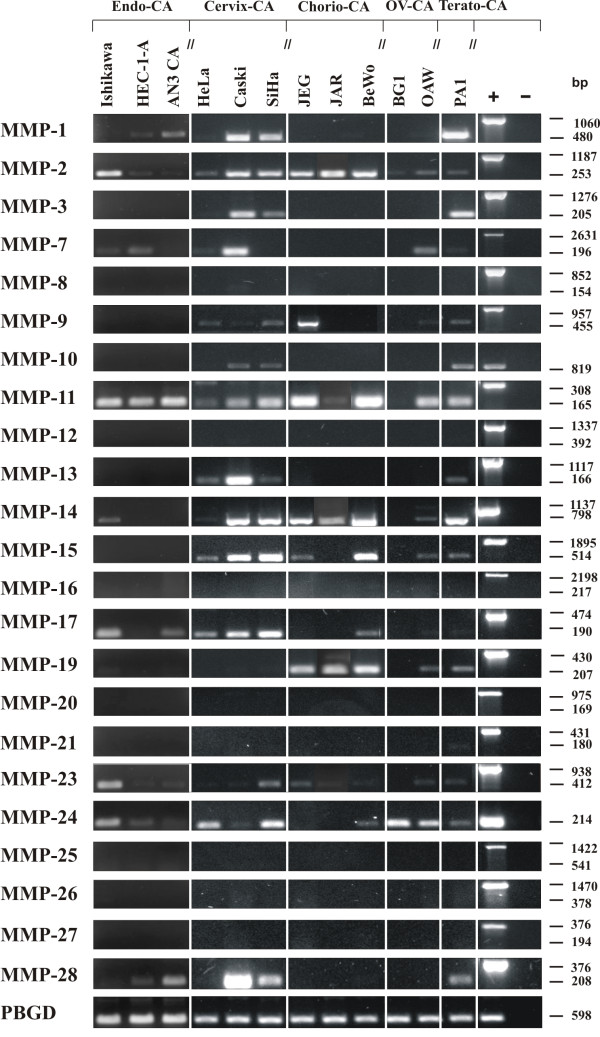
**MMP pattern in human gynecological cancer cell lines analyzed by semiquantitative RT-PCR**. Total mRNA from the folowing gynecological cancer cell lines was extracted and used as template for RT-PCR analysis: endometrium carcinoma (Endo-CA), cervical carcinoma (Cervix-CA), chorion carcinoma (Chorio-CA), ovarian carcinoma (OV-CA) and teratocarcinoma (Terato-CA). Primers, specific for each transcript were designed in flanking exons (for primer deteils see [46]), resulting in longer amplicons if human genomic DNA was amplified (positive control (+)) and in shorter amplicons representing cDNAs. The housekeeping gene PBGD was used as internal loading control and amounts of cDNA were normalized to the amount of PBGD for each sample.

**Figure 2:**
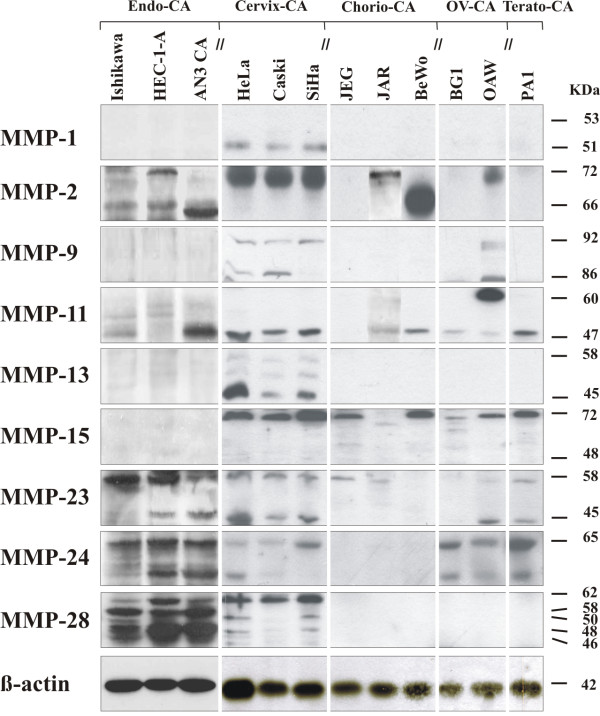
**Protein expression of MMPs in different human gynecological cancer cell lines as analyzed by Western blot**. Protein lysates were isolated from the gynecological cancer cell lines and separated by polyacrylamid gel electrophoresis. Expressed MMP proteins were visualized using specific antibodies, capable of recognizing both, the inactive and active, smaller forms of MMPs (antibodies are summarized in Table 2). β-actin was used as internal loading control.

**Figure 3:**
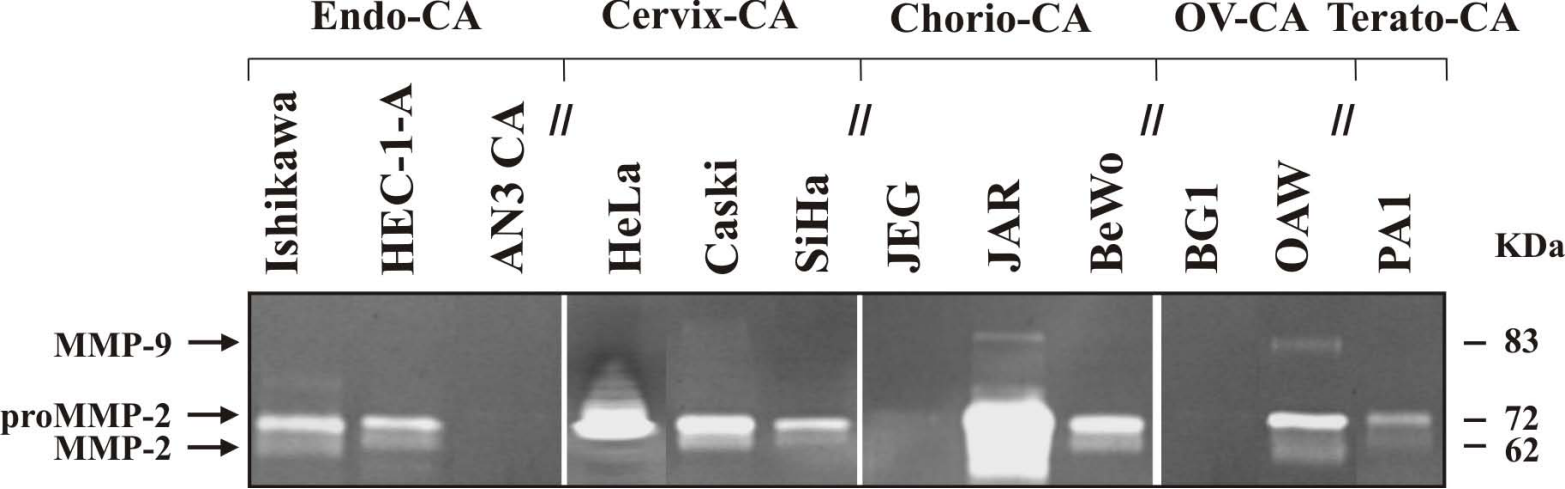
**Analysis of the cell culture supernatants by gelatin zymography**. The cell lines were first plated in serum-containing medium for 72 h. Afterwards, medium was replaced by serum-free DMEM/HamsF12 for an additional 48 h. Samples of conditioned medium were assayed for MMP-2 and MMP-9 by gelatin zymography. Gelatinolytic activity of pro and active MMP-2 and active MMP-9 are visible as a clear area on the gel, indicating where the gelatine has been digested.

A varying expression pattern of MMPs could be observed on an mRNA level, depending on the cell line investigated. Except for MMP-16, -20, -25, -26 and -27, mRNA could be detected for all other MMPs in at least one of the cell lines. For MMP-8, -12 and -21 only very weak mRNA expression could be observed in single cell lines. Nine MMPs, which were present in most of the cell lines, were chosen for further expression analysis on protein level. The results of the semiquantitative RT-PCR and Western blot are summarized in Figures 1 and 2. The results of the densitometrically quantified expression of the mRNAs and proteins are shown in Table 3 and 4, respectively. The enzymatic activity of two gelatinases (MMP-2 and -9) in the serum-free cell culture supernatants was examined by gelatin zymography and the corresponding data is presented in Figure 3.

**Table 3:** Expression levels of MMP mRNA in gynecological cancer cell lines

	Ishikawa	HEC-1-A	AN3 CA	Hela	Caski	SiHa	JEG	JAR	BeWo	BG-1	OAW-42	PA1
**MMP1**	0	(+)	+	(+)	+++	+++	0	0	0	0	(+)	+++
**MMP2**	+++	+	(+)	+	+++	+++	+++	+++	+++	(+)	+	+
**MMP3**	0	0	0	(+)	+++	++	0	0	0	0	0	+++
**MMP7**	(+)	+	(+)	(+)	+++	0	0	0	0	0	++	(+)
**MMP8**	0	0	0	0	(+)	0	0	0	0	0	0	0
**MMP9**	0	0	0	(+)	(+)	+	+++	0	0	0	(+)	+
**MMP10**	0	0	0	0	+	+	0	0	0	0	0	++
**MMP11**	++	++	++	+	++	++	+++	+	+++	(+)	++	++
**MMP12**	0	0	0	0	(+)	0	0	0	0	0	0	0
**MMP13**	0	0	0	+	+++	+	0	0	0	0	0	+
**MMP14**	+	0	0	(+)	+++	+++	+++	++	+++	0	+	+++
**MMP15**	0	0	0	+	+++	+++	+	0	+++	0	+	+
**MMP16**	0	0	0	0	0	0	0	0	0	0	0	0
**MMP17**	++	0	+	+	++	++	0	0	+	0	(+)	(+)
**MMP19**	(+)	0	0	0	0	0	+++	+++	+++	0	+	++
**MMP20**	0	0	0	0	0	0	0	0	0	0	0	0
**MMP21**	0	0	0	0	0	0	0	0	0	0	0	(+)
**MMP23**	++	0	0	(+)	(+)	++	+	(+)	(+)	0	(+)	(+)
**MMP24**	++	+	(+)	++	(+)	++	0	0	+	++	++	+
**MMP25**	0	0	0	0	0	0	0	0	0	0	0	0
**MMP26**	0	0	0	0	0	0	0	0	0	0	0	0
**MMP27**	0	0	0	0	0	0	0	0	0	0	0	0
**MMP28**	(+)	+	++	(+)	+++	++	0	0	0	0	0	++

Scored from 0 = no expression, (+) = very weak expression, + = weak expression, ++ = moderate expression to +++ = high expression

**Table 4:** Expression of MMP proteins in different gynecological cancer cell lines

	Ishikawa	HEC-1-A	AN3 CA	Hela	Caski	SiHa	JEG	JAR	BeWo	BG-1	OAW-42	PA1
**proMMP1**	0	0	0	0	0	0	0	0	0	0	0	0
**MMP1**	0	0	0	+	+	+	0	0	0	0	0	0
**proMMP2**	(+)	+	(+)	+++	+++	+++	0	+	0	0	++	0
**MMP2**	+	+	++	0	0	0	0	0	+++	0	0	0
**proMMP9**	0	0	0	+	+	+	0	0	0	0	+	0
**MMP9**	0	0	0	+	++	0	0	0	0	0	++	0
**proMMP11**	0	(+)	(+)	0	0	0	0	+	0	0	+++	0
**MMP11**	+	0	+++	++	+	++	0	+	+	(+)	(+)	+
**proMMP13**	0	0	0	(+)	(+)	(+)	0	0	0	0	0	0
**MMP13**	0	0	0	+++	(+)	++	0	0	0	0	0	0
**proMMP15**	0	0	0	++	++	+++	+	0	++	(+)	+	++
**MMP15**	0	0	0	(+)	0	0	0	0	0	0	0	0
**proMMP23**	+++	+++	++	++	+	+	+	(+)	0	0	(+)	+
**MMP23**	(+)	++	++	+++	+	++	0	0	0	0	++	+
**proMMP24**	+	++	++	+	+	++	0	0	0	++	+	+++
**MMP24**	(+)	++	++	+	(+)	0	0	0	0	+	+	+
**MMP28(62)**	+	+++	++	++	++	+++	0	0	0	0	0	0
**MMP28(58)**	+++	+++	+++	0	0	0	0	0	0	0	0	0
**MMP28(50)**	+	0	0	+	0	+	0	0	0	0	0	0
**MMP28(48)**	++	+++	+++	+	0	+	0	0	0	0	0	0
**MMP28(46)**	+	++	++	(+)	0	(+)	0	0	0	0	0	0

Scored from 0 = no expression, (+) = very weak expression, + = weak expression, ++ = moderate expression to +++ = high expression

### Expression of MMPs in endometrial cancer cell lines

In the Ishikawa cell line, the highest expression was detected for MMP-2 and -11 on an mRNA level, but only a weak expression of their proteins could be observed in the cell lysates. However, moderate gelatinolytic activity of the secreted latent form of MMP-2 could be identified by gelatin zymography, whereas its active form showed very weak activity. For MMP-23, moderate mRNA and strong expression of its inactive protein was seen in the same cell line. Albeit the highest expression of MMP-24 mRNA was detected in Ishikawa cells, on Western blot its expression was weaker compared to other two endothelial cancer cell lines. In Ishikawa, the expression of MMP-28 both on mRNA as well as on protein level was weaker compared to the two other endothelial cancer cell lines. In addition, MMP-7, -14, -17 and -19 were detected in the Ishikawa cells by RT-PCR only.

Although the highest expression of MMP-11 mRNA was identified in the HEC-1-A cells, no protein expression was detectable in this cell line. The same cell line showed a weak expression of MMP-2 on mRNA level, a moderate expression on protein level as well as corresponding gelatinolytic activity of its secreted protein. Even though the expression of MMP-23 mRNA was the weakest among endometrial cancer cell lines, for its inactive form as well as active protein strong expression was observed. A high expression of proteins of approximately 65 KDa and 55 KDa could be identified for MMP-24 in HEC-1-A cells, whereas for MMP-28 a strong expression of three protein bands of approximately 62 KDa, 58 KDa and 48 KDa could be seen. Additionally, very weak expression of MMP-1 and -7 could be also detected in this cell line, but only on mRNA level.

The highest expression of active forms of MMP-2 and -11 proteins among the three examined endometrial cell lines was detected in AN3 CA cells, although for MMP-2 only a weak mRNA expression could be identified. In this cell line, MMP-23 showed similar mRNA and protein expression patterns like in HEC-1-A. For MMP-24 and -28 the expression was detected on both, mRNA and protein level, whereas for MMP-1 and -17 only mRNA could be identified.

### Expression of MMPs in cervical cancer cell lines

The majority of the analyzed MMPs could be identified in all three cervical cell lines examined by RT-PCR. While for MMP-2 a moderate to strong expression of its mRNA could be found in HeLa, Caski and SiHa cells, on protein level a very strong expression of its inactive form was detected by Western blot. In addition, using gelatin zymography we showed that all three of these cultivated cell lines were secreting corresponding amount of the latent form of MMP-2 in serum-free medium. Furthermore, MMP-1, -3, -7, -8, -9, -11, -13, -14, -15, -17, -23 and -24 all showed diverse expression levels of their mRNAs with the highest expression level in the Caski cell line. Active protein forms of MMP-1 and -11, inactive protein form of MMP-15, and both inactive and active MMP-9, -13 and -23 were observed on Western blot. For MMP-24, we were able to detect a band of approximately 65 KDa in Caski and an additional band of approximately 55 KDa in HeLa cells. Lastly, all three cervical cancer cell lines four protein bands of approximately 62, 50, 48 and 46 kDa were found for MMP-28.

### Expression of MMPs in chorioncarcinoma cell lines

Albeit a clear expression existed of MMP-2, -9, -11, -14 and -19 mRNAs in the JEG cell line, their proteins could not be detected using Western blot analysis. The only proteins found in this cell line were the latent forms of MMP-15 and -23 at moderate levels corresponding to the expression of their mRNAs.

A strong expression of MMP-2 mRNA was detected in JAR cells. Extremely robust gelatinolytic activity of its secreted protein could be identified in the serum-free medium, whereas on Western blot only a moderate protein expression of the inactive form could be seen in the cells. Active MMP-9 showed very weak gelatinolytic activity, although on Western blot no expression could be seen. Weak expression of both mRNAs and inactive protein forms of MMP-11 and -23 could also be identified in this cell line. In addition, expression of MMP-14 and -19 was detected but only on mRNA level.

The highest expression found in all cell lines tested of the active protein forms of MMP-2 and -11 was detected in BeWo cells. Gelatin zymography also revealed activity of MMP-2 secreted by BeWo cells. For MMP-15, a strong expression of its mRNA was present but the latent protein form could only be detected in those cells. Further, solely MMP-14, -17, -19 and -24 could be identified by RT-PCR only.

### Expression of MMPs in ovarian and teratocarcinoma cell lines

A strong expression of the mRNA and protein (approximately 65 KDa and 55 KDa) of MMP-24 was found in the ovarian carcinoma derived BG1 cells. Rather, a weak expression of MMP-2 and -11 was also seen on Western blot in this cell line.

For MMP-2, -15 and -24, a moderate expression of mRNAs and latent protein forms were detected in the OAW-42 cell line. Regarding OAW-42 cells, MMP-11 showed strong expression of its inactive protein whereas for MMP-9 and -24 moderate expressions of both inactive and active proteins were identified. Zymographic analysis of the serum-free cell culture supernatant identified strong gelatinolytic activity of latent MMP-2 as well as weak activity of active MMP-9. Additionally, expression of MMP-7, -14 and -19 was detected on a mRNA level.

The highest expression was detected for MMP-1 on mRNA level in the teratocarcinoma cell line PA-1 but no corresponding protein expression could be detected by Western blot analysis. Secreted MMP-2 showed weak gelatinolytic activity. For MMP-11 moderate mRNA and protein expression was seen in this cell line and moderate expression of MMP-15 mRNA and inactive protein form could be observed herein, whereas for MMP-23 a weak expression could be observed by RT-PCR and Western blot. Although only a weak expression of MMP-24 mRNA was detected in PA-1 cells, a strong expression of two protein bands of 65 KDa and approximately 55 KDa were seen in Western blot. The PA-1 cell line was the only one amongst the investigated cell panel which showed a weak PCR product for MMP-21.

## Discussion

Degradation of the extracellular matrix is a condition for invasive growth of malignant tumors. Metalloproteinases (MMPs) play a very important role in this process. The role and the contribution of the tumor and stromal cell compartments to the increased levels of MMPs in carcinoma tissue are still poorly understood. Some investigators suggest an almost exclusive stromal origin of MMPs detected in cancer tissue [1]. Other studies demonstrate that a lot of MMPs are constitutively expressed in several tumor cell lines in the absence from any stromal component [18]. Our objective was to investigate which MMPs are expressed in different gynecological cancer cell lines and thus to identify useful model system for further analysis on MMP regulation in cancer.

MMP-2, -7 and -9 were found to be expressed in uterine serous carcinoma as well as in endometrioid carcinoma of the uterus by immunohistochemistry [49]. The endometrial carcinoma derived cell line Ishikawa was shown to secrete MMP-1, -2 and -9 [50]. However in our Ishikawa cell line, mRNA and protein could be detected for MMP-2 but not for MMP-1 and -9, which could be influenced by different primers used or different cell culture conditions that might affect MMP expression. MMP-1 was described in HEC-1-A and AN3 CA cells [24] and in those cell lines we found a corresponding expression of its mRNA. However, no expression could be identified for MMP-1 protein in those endometrial cell lines. Our results confirm those of Park et al., who did not detect MMP-9 mRNA in HEC-1-A cells using RT-PCR [51]. Whereas in contrast to our negative findings by Western Blot, MMP-1, -2, -7, -9 and -14 protein could be detected in HEC-1-A cells using immunohistochemistry by Tanaka [52]. These differences might be due to different culture conditions or primers and antibodies (and techniques - WB versus immunohistochemsitry) used. Also, mRNA stability of MMP transcripts contributes to the metalloproteinase product amount. There is evidence about the regulation of the MMP-9 mRNA stability by α3β1 integrin, among others, that is associated with mammary carcinoma cell metastasis and invasion [53,54]. Modulation of its mRNA stability might be important during malignant conversion and metastasis, when tumor cells need to induce or maintain MMP-9 levels in response to changing environmental cues. In endometrial cancer, a high expression of MMP-2 and low expression of TIMP-2 seem to be potent markers for tumors, which provide a high risk of local and distant metastasis [55]. In our study MMP-2 mRNA as well as MMP-2 protein was found in all three endometrial cancer cell lines. We also identified moderate gelatinolytic activity of MMP-2 protein that was secreted by Ishikawa and HEC-1-A cells. Expession analysis of TIMPs, including TIMP-2, remains to be done however in our endometrial cancer cell lines. A relation between higher expression of MMP-2 and -9 and progression of endometrial carcinoma was detected by Di Nezza et al. using in situ hybridization and in situ zymography. MMP-2, -9 and -14 were mainly localized in epithelial tumor cells, whereas only a variable stromal localization could be detected [56]. They also found a co-localization of MMP-14 with MMP-2, supporting the role of MMP-14 in the activation of pro-MMP-2. In our cell lines, only Ishikawa was found positive for MMP-14 mRNA. However, protein detection in the Western blot was not possible by the antibodies available. Maximum levels of MMP-26 mRNA were found in normal endometrial tissue and in endometrial hyperplasia, whereas the amount of MMP-26 mRNA was downregulated in all malignant samples investigated [57]. Consequently, in our study none of the tested endometrial cancer cell lines was positive for MMP-26 mRNA. This finding further fits to the data by Isaka and co-workers, where all but one endometrial tissue sample as well as all endometrial cancer cell lines including HEC-1-A were negative for MMP-26 mRNA [58]. In contrast to our results, as we found a weak expression of MMP-7 mRNA in HEC-1-A cells, they did not detect MMP-7 mRNA in this cell line. This difference might be due to either different primers or conditions used, or to different cell culture conditions that may influence MMP expression [59]. To the best of our knowledge, there are no available data in literature about the expression of the other MMPs in endometrial cancer cell lines. According to our results, the Ishikawa cell line showed the broadest range of mRNA and protein expression of most of the MMPs analyzed and thus could be the best choice as model cell line for future experiments on the role of MMPs in endometrial carcinoma development and as a positive control for MMP research. The expression of MMP-11, -23, -24 and -28, which was identified in our study on both, mRNA and protein level, could be related to the development of endometrial carcinoma and awaits further investigation in this cancer entity. Remarkably, the expression of MMP-23 protein was however on higher level compared to its mRNA, which might be due to increased efficiency of MMP-23 translation in endometrial cancer. Using the antibody for MMP-28 we detected bands of approximately 62, 58, 50, 48 and 46 kDa. However, we did not have enough data to discriminate inactive and active forms of this protein since there is barely any information about its protein size. At least three MMP-28 transcripts of 2.6, 2.0, and 1.2 kb have been reported representing alternatively spliced forms, differentially expressed in human tissues [60] and isoforms which encode proteins of 520 and 393 amino acids with predicted respective masses of 58.9 and 44.5 kDa.

In the cervix, it was shown that MMP-2, -3 and -9 are present in the tissue of cervical adenocarcinomas, whereas no expression of these MMPs could be detected in the nonneoplastic endocervical epithelium [10]. In accordance to this, Wang et al. detected a higher expression of MMP-2 mRNA in cervical carcinomas then in normal counterparts of the uterine cervix [12] and we found MMP-2 mRNA in all three cervical carcinoma derived cell lines as well. We also found a strong expression of inactive MMP-2 in those cells using Western blot as well as a strong gelatinolytic activity of its secreted protein. The high expression of MMP-14 described by Zhai and colleagues in tissues of cervical carcinomas corresponds to our finding of strong mRNA expression in Caski and SiHa cell lines [13]. Further, we found a strong expression of MMP-15 mRNA in those cell lines. These results are in line with results obtained by Iwasaki et al. [19]. In contrast to our study Iwasaki and co-workers did not detect MMP-1 in Caski or SiHa cells, whereas we found a strong expression of MMP-1 mRNA and a weak expression of active MMP-1 in both cell lines. In HeLa cells, only few MMPs were expressed at lower amounts. Taken together the identified expression profile leads to the conclusion that future experiments on invasion of cervical cancer cells would be promising using Caski or SiHa cells as a model. In addition, since MMP-1, -11, -13, -15, -17, -24 and -28 are expressed in all three cervical carcinoma cell lines analyzed, these could be good candidates for further expression analysis in cervical carcinoma tissues as well.

To our knowledge, there are just few amount of data available about the expression of MMPs in chorioncarcinoma cell lines. In the JEG cell line we detected MMP-2, -9, -11, -14, -15, -19 and -23 mRNA whereas on protein level only weak expression of latent MMP-15 and -23 was observed. Giambernardi et al. also investigated the expression of the abovementioned MMPs in JEG cells and observed the expression of MMP-12 (which was negative in our results) and -14, but not the expression of the remaining MMPs [18]. These differences may be due to some variations in cell culture conditions (e.g. differences in serum containing growth factors added to the culture medium). We found a moderate to strong expression of MMP-2, -11, -14, -15 and -19 mRNA in BeWo cells, whereas on protein level only proMMP-15 and active MMP-2 and -11 were detectable. In addition, our zymography analysis of secreted MMP-2 identified moderate gelatinolytic activity of its latent and active forms. These differences in the expression pattern between mRNA and protein level might be due to regulation of the translational level [61]. In line with our results, the expression of MMP-2 was already described in BeWo cells [62]. Our data about the expression pattern of MMPs in the JAR cell line showed a week to moderate expression of MMP-2, -11, -14, -19 and -23 on mRNA level, but only a weak expression of MMP-11 and -23 protein could be identified. However, for MMP-2 we were able to detect protein expression in the cells as well as very strong gelatinolytic activity of its secreted protein. Thus, based on our analysis, we suggest BeWo cells as the best model for future analyses of MMP biology and regulation in chorioncarcinoma cell lines.

In ovarian cancer, MMP-2 and -9 seem to be expressed more frequently in early lesions than in established carcinomas [14]. Overexpression of MMP-2, -9 and -14 seems to also prepare the ground for development and growth of malignant ovarian tumors [16]. According to these findings, MMPs might play a critical role in the first steps of tumorigenesis in ovaries. Surprisingly, to our knowledge, no single study to date investigated the expression of MMPs in the ovarian cancer cell lines OAW-42, BG-1 and in the teratocarcinoma cell line PA-1 compared to already performed examinations of endometrial, cervix or choriocarcinoma cancer cell lines, as already discussed. The PA-1 cells do express a relatively broad range of 15 different MMP-RNAs. While on mRNA level only a weak expression of MMP-15 and -24 could be observed a moderate to strong expression of pro-MMP-15 and -24 proteins was detectable. Further, the active form of MMP-11 and both, inactive and active forms of MMP-23 were detected. OAW-42 cells showed a remarkable high expression of MMP-11 as mRNA and protein. Further, mRNAs and proteins of MMP-2, -9, -15 and -23 were moderately expressed in this cell line. According to this finding we also detected gelatinolytic activity of secreted MMP-2 and MMP-9 by performing zymography analysis of the cell culture supernatant. Based on our data, there are many more MMPs beside the commonly investigated MMP-2, -9 and -14, which are expressed in ovarian cancer cell lines and are thus candidates for future analyses on their influence on the development of ovarian cancer.

In our study we could not detect the mRNAs of MMP-12, -16, -20, -25, -26 and -27 in any of the twelve cell lines analyzed. However due to the genomic DNA control and the positive other MMPs in the same preparations, we could ascertain that the RT-PCR itself worked. Concerning MMP-20, these results are in line with results obtained by Giambernardi et al. who also did not detect MMP-20 in any of the eighty-four cell lines analyzed in their study [18].

In summary, we detected a broad and diverse expression pattern of MMPs in different cell lines representing different human gynecological cancer entities. Our data indicate that there is no real pattern of MMP expression related to cancer type or metastasis. Even within the same cancer stage MMPs have a diverse expression, as our previous analysis of breast cancer and glioblastoma showed [46,63]. Therefore, further studies on MMPs and a better understanding of their role in tumor invasion and metastasis are necessary. The results presented here could establish thus a basis for the analysis of the regulation of MMP expression in gynecological tumors, which could be performed in these cell lines selected as a model system.

## Conclusions

This study demonstrates that gynecological cell lines grown in vitro and therefore being independent of environmental factors can constitutively express a wide variety of MMPs on mRNA and protein level. MMP-2, -11, -14 and -24 are found in most of the cell lines analyzed. MMP-1 and -7 were expressed in all but chorioncarcinoma cells, whereas MMP-9 and -15 showed the same expression pattern concerning endometrial cancer cell lines. In addition, MMP-3, -10 and -13 were expressed in cervical carcinoma and teratocarcinoma cell lines only.

Caski and PA-1 cell lines could be the best choice for all future experiments on the regulation of MMPs and their role in gynecological cancers. Additionally, the PA-1 cell line showed the strongest mRNA and protein expression of most of the MMPs analyzed and therefore could be used as the positive control for their expression analysis in general. These cell lines are also promising candidates for future investigations dealing with the role of MMPs in tumor invasion and building of metastatic formations. Although expression on mRNA and protein level was quite less in comparison to the abovementioned cell lines, BeWo cells could be the best choice for future experiments concerning chorioncarcinoma cell lines and the Ishikawa cell line concerning endometrial carcinoma, whereas OAW could be used for the ovarial cancer analysis.

## List of abbreviations

bp: base pare; DTT: dithiothreitol; ER: estrogen receptors; hCG: human chorionic gonadotropin; HCS: human chorionic somatomammotropin; HPV: human papillomavirus; kDa: kilodalton; MMPs: matrix metalloproteinases; PBGD: porphobilinogen deaminase; PBS: phosphate-buffered saline; PR: progesterone receptors; RT: reverse transcriptase; U: unit

## Competing interests

The authors declare that they have no competing interests.

## Authors' contributions

AS drafted the manuscript, set up the experiments, collected the data, analyzed and interpreted the results. UK participated in the study design, interpretation of the results and finalization of the manuscript. SF and MK carried out the PCR and Western Blot analysis. JD participated in editorial support. JA participated in the study design, experimental concept, interpretation of the results and drafting of the manuscript. All authors read and approved the final manuscript.

## Pre-publication history

The pre-publication history for this paper can be accessed here:

http://www.biomedcentral.com/1471-2407/10/553/prepub

## Supplementary Material

Additional file 1 **MMP expression in gynecological cancer cell lines**. Click here for file
