# Supplementary material for: Expression pattern of matrix metalloproteinases in human gynecological cancer cell lines
Source: BMC Cancer. 2010 Oct 13;10:553. doi: 10.1186/1471-2407-10-553 (PMC2964638; doi:10.1186/1471-2407-10-553)
Supplement: Additional file 1 — MMP expression in gynecological cancer cell lines. [file 1471-2407-10-553-S1.DOC]

**Additional File 1**: Expression of MMP mRNA and protein in endometrial, cervix and chorioncarcinoma cell lines

|  | **HEC-1-A** | **HeLa** | **Caski** | **SiHa** | **JEG** | **JAR** | **BeWo** |
| --- | --- | --- | --- | --- | --- | --- | --- |
| **MMP1** | + Tanaka et al. [52] | - Giambernardi et al. [18] | - Iwasaki et al. [19] | - Iwasaki et al. [18] | - Giambernardi et al. [18] | n.a. | n.a. |
| **MMP2** | + Tanaka et al. [52]  - Park et al. [51] | - Giambernardi et al. [18] | + Silva Cardeal et al. [64]  + Iwasaki et al. [19]  + Kato et al. [65] | + Mitra et al. [66]  + Iwasaki et al. [19]  + Silva Cardeal et al. [64]  + Kato et al. [65] | + Zhang et al. [65]  + Giambernardi et al. [18] | + DiSimone et al. [68] | + Morgan et al.[62] |
| **MMP3** |  | - Giambernardi et al. [18] | n.a. | n.a. | - Giambernardi et al. [18] | n.a. | n.a. |
| **MMP7** | + Tanaka et al. [52]  - Isaka et al. [58] | + Giambernardi et al. [18] | n.a. | n.a. | - Giambernardi et al. [18] | n.a. | n.a. |
| **MMP8** |  | - Giambernardi et al. [18] | n.a. | n.a. | - Giambernardi et al. [18] | n.a. | n.a. |
| **MMP9** | + Tanaka et al. [52]  - Park et al. [51] | - Giambernardi et al. [18] | + Silva Cardeal et al. [64]  + Kato et al. [65]  - Iwasaki et al. [19] | + Mitra et al. [66]  - Silva Cardeal et al. [64]  - Iwasaki et al. [19] | + Zhang et al. [67]  - Giambernardi et al. [18] | n.a. | - Morgan et al.[62] |
| **MMP10** |  | - Giambernardi et al. [18] | n.a. | n.a. | - Giambernardi et al. [18] | n.a. | n.a. |
| **MMP11** |  | - Giambernardi et al. [18] | n.a. | n.a. | - Giambernardi et al. [18] | n.a. | n.a. |
| **MMP12** |  | - Giambernardi et al. [18] | n.a. | n.a. | + Giambernardi et al. [18] | n.a. | n.a. |
| **MMP13** |  | - Giambernardi et al. [18] | n.a. | n.a. | - Giambernardi et al. [18] | n.a. | n.a. |
| **MMP14** | + Tanaka et al. [52] | + Giambernardi et al. [18] | + Iwasaki et al. [19]  + Silva Cardeal et al. [64] | + Iwasaki et al. [19]  + Silva Cardeal et al. [64] | + Giambernardi et al. [18] | n.a. | n.a. |
| **MMP15** |  | + Giambernardi et al. [18] | + Iwasaki et al. [19] | + Iwasaki et al. [19] | + Giambernardi et al. [18] | n.a. | n.a. |
| **MMP16** |  | + Giambernardi et al. [18] | - Iwasaki et al. [19] | - Iwasaki et al. [19] | - Giambernardi et al. [18] | n.a. | n.a. |
| **MMP17** |  | + Giambernardi et al. [18] | n.a. | n.a. | - Giambernardi et al. [18] | n.a. | n.a. |
| **MMP20** |  | - Giambernardi et al. [18] | - Giambernardi et al. [18] | - Gimbernardi et al. [17] | - Giambernardi et al. [18] | - Giambernardi et al. [18] | - Giambernardi et al. [18] |
| **MMP26** | - Isaka et al. [58] | n.a. | n.a. | n.a. | + Zhang et al. [67] | n.a. | n.a. |

+ = expression; - = no expression, n.a. = not analysed
